# Molecular Insights into the Genetic Variability of ORF Virus in a Mediterranean Region (Sardinia, Italy)

**DOI:** 10.3390/life11050416

**Published:** 2021-05-04

**Authors:** Elisabetta Coradduzza, Daria Sanna, Angela M. Rocchigiani, Davide Pintus, Fabio Scarpa, Rosario Scivoli, Roberto Bechere, Maria A. Dettori, Maria A. Montesu, Vincenzo Marras, Renato Lobrano, Ciriaco Ligios, Giantonella Puggioni

**Affiliations:** 1 Istituto Zooprofilattico Sperimentale della Sardegna, 07100 Sassari, Italy; elisabetta.coradduzza@izs-sardegna.it (E.C.); angelamaria.rocchigiani@izs-sardegna.it (A.M.R.); davide.pintus@izs-sardegna.it (D.P.); rosario.scivoli@izs-sardegna.it (R.S.); roberto.bechere@izs-sardegna.it (R.B.); mariaantonietta.dettori@izs-sardegna.it (M.A.D.); ciriaco.ligios@izs-sardegna.it (C.L.); giantonella.puggioni@izs-sardegna.it (G.P.); 2 Dipartimento di Scienze Biomediche, Università di Sassari, 07100 Sassari, Italy; 3 Dipartimento di Medicina Veterinaria, Università di Sassari, 07100 Sassari, Italy; fscarpa@uniss.it; 4 Dipartimento di Scienze Mediche, Chirurgiche e Sperimentali, Università di Sassari, 07100 Sassari, Italy; mmontesu@uniss.it (M.A.M.); vincenzo.marras@aousassari.it (V.M.); renato.lobrano@gmail.com (R.L.)

**Keywords:** contagious ecthyma, phylogenetic analysis, molecular dating, VIR, B2L, O45, sequencing

## Abstract

Orf virus (ORFV) represents the causative agent of contagious ecthyma, clinically characterized by mild papular and pustular to severe proliferative lesions, mainly occurring in sheep and goats. In order to provide hints on the evolutionary history of this virus, we carried out a study aimed to assess the genetic variation of ORFV in Sardinia that hosts a large affected small ruminant population. We also found a high worldwide mutational viral evolutionary rate, which resulted, in turn, higher than the rate we detected for the strains isolated in Sardinia. In addition, a well-supported genetic divergence was found between the viral strains isolated from sheep and those from goats, but no relevant connection was evidenced between the severity of lesions produced by ORFV and specific polymorphic patterns in the two species of hosts. Such a finding suggests that ORFV infection-related lesions are not necessarily linked to the expression of one of the three genes here analyzed and could rather be the effect of the expression of other genes or rather represents a multifactorial character.

## 1. Introduction

Orf virus (ORFV; Family: Poxviridae) is the causative agent of contagious ecthyma (ORF) and is the prototype of the genus *Parapoxvirus* [1], whose representatives are bovine papular stomatitis virus (BPSV), *Pseudocowpox* virus (PCPV), and *Parapoxvirus* of red deer in New Zealand (PVNZ). ORFV causes patterns of lesions (ranging from mildly papular and pustular to severely proliferative) that occur primarily in sheep and goats, but similar lesions have also been reported in other animal species [2] and humans [1,3], resulting in zoonotic disease. ORFV was reported for the first time in Southwest Africa by Zeller in 1920 [4], and it has now spread all over the world [5]. In ruminants, the lesions provoked by the disease appear mainly on the mouth, muzzle, nostrils, gums, and tongue; they occasionally appear in the feet and udders and can extend to the gastrointestinal tract and respiratory apparatus [6]. The infection affects mainly lambs and kids, sporadically causing death since the lesions caused by viral infection prevent them from sucking milk. The degree of morbidity of the disease is very high, and although mortality is generally rare, it can reach up to 10% in lambs [7] and 93% in kids [8,9]. In humans, the main individuals at risk are those in contact with livestock, such as farmers, veterinarians, hunters, and butchers [1]. Human lesions are located mainly on the hands and undergo spontaneous resolution in a few weeks, showing a benign course; however, malignant cases have also been reported with atypical proliferative lesions, particularly in immunocompromised individuals [2,10,11].

The virus spreads and acts mainly in late summer as it is favored by hot, humid, and dry environments, while its resistance is reduced if the climate is cold or rainy [10,11,12]. The impact of ORFV on the individual is correlated with age, immune status (immune defects or persistent infection), breed of hosts, and the hygienic condition of the farms [10,12]. The transmission of the virus is very rapidly mediated by direct contact through damaged skin or contaminated fomites [12].

The genome of ORFV is a linear, double-stranded 135 kb-long DNA molecule encoding 132 genes [2,13]. The central region contains highly conserved genes involved in viral replication and the genesis of the viral structure, while terminal regions are more variable and contain genes for virulence and immunomodulation [2,14]. The high nucleotide variability of terminal regions is likely involved in the phenomenon of reinfection that is typical of ORFV, which also occurs as a product of regulation by viral genes in the host’s innate immune response [10].

Molecular analysis of ORFV has been performed on strains from almost all continents (i.e., [5,15]). These studies were generally based on the sequencing of the major envelope gene (B2L) but also on the analysis of the viral interferon resistance (VIR) and late transcription factor VTLF-1 (O45) genes. To date, only a few studies have focused on the genetic variation of ORFV from the Italian peninsula [16,17,18,19], while no molecular data have been reported for the region of Sardinia, where a large population of small ruminants is present. This island could be historically considered an endemic area for ORFV spread due to the high density of host species.

In this context, we provide the first study aimed at assessing the genetic variation of ORFV in Sardinia. This Mediterranean island can be used as an ideal model for the study of the evolutionary history and spreading dynamics of this virus. Indeed, Sardinia represents an enclosed system in which viruses can potentially evolve independently with reduced contributions from external gene pools, thus originating private haplotypes that are absent outside the island, as also recently reported for the African swine fever virus by Franzoni et al. [20]. For these reasons, the study of ORFV in Sardinian isolates could provide new scenarios and clues on the general behavior of this virus. Here, the analysis of three viral genes isolated from animal and human samples will be used to characterize the strains that spread in Sardinia to depict the genetic structuring present on the island and shed some light on its possible connection with the species of hosts and severity of lesions produced by viral infection.

Furthermore, we also provide the first inference on the phylogenetic patterns of ORFV from a global perspective, providing new results regarding the evolutionary history of this virus. We reached this goal by analyzing the variation of the VIR gene, which is considered one of the best molecular markers used to date for genetic characterization in the genus *Parapoxvirus* [21,22].

## 2. Materials and Methods

### 2.1. Sampling

**Figure 1:**
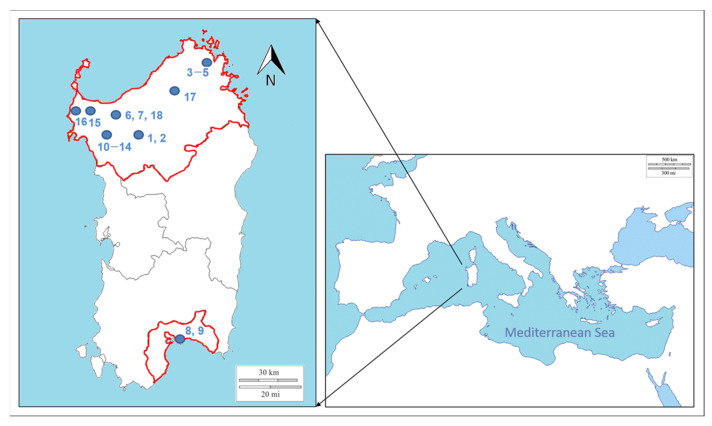
Sampling plan. The geographic locations of the farms where samples were collected are indicated in the map of the Mediterranean island of Sardinia. The numbers at each site refer to the sample codes as reported in Table 1. The hyphen between two numbers indicates an interval. Blue spots indicate the municipalities where the farms were located, as reported in Table 1.

Sampling was performed between March 2017 and December 2019 in 6 different Lacaune and Sarda breed sheep flocks and in 2 Sarda goat herds (Figure 1). At each farm, newborn lambs and kids, and adult animals showed clinical signs of ORFV infection, that were also confirmed by virological and molecular analyses.

Clinical ORF outbreaks were reported by the private practitioner and veterinary public health professionals involved in our research during their diagnostic activity. This sampling did not provide epidemiological indications regarding the incidence and geographical distribution of ORFV on the island.

Tissue samples from different skin-damaged areas were collected for histopathological and virological investigations from 9 affected sheep and 8 affected goats of different ages (see Table 1 for details). In addition, biopsy samples were also collected for histological and virological investigations from skin from the hand of a young ORFV-affected farmer who showed papular and pustular lesions. The case of this farmer was reported by the dermatological clinic of Sassari when the outbreak on the farm where he became infected had already passed.

Upon collection, samples were stored at −20 °C until DNA extraction. A subset of the same samples was fixed in 10% formalin to be used for histopathological analyses.

### 2.2. Histopathology

ORF lesions of the skin and buccal mucosae of hosts were sampled (including a portion of normal adjacent tissue) and promptly fixed in 10% neutral buffered formalin.

Fixed tissues were processed using a sequence of a series of alcohol solutions in ascending concentrations up to 100% to replace an aqueous environment with a hydrophobic environment.

Then, the samples were clarified in organic solvent (xylene) to remove the alcohol prior to infiltration of the specimen with melted paraffin. Finally, specimens were embedded in a block of wax for section cutting. Paraffin blocks were cut into 4 µm-thick slices, dewaxed using xylene and descending concentrations of alcohol, and finally stained using the ST Infinity Haematoxylin & Eosin Staining System (Leica Biosystem Richmond, Richmond, IL, USA). This routine staining method is used to reveal a comprehensive picture of different tissue structures and conditions.

Stained sections were covered with cover glasses and examined under a light microscope with a magnification range extending from ×50 to ×400.

### 2.3. Virus Isolation

For each sample, a 0.5 g piece of tissue (see Table 1) was homogenized using a tissue homogenizer in 5 mL (10% *w*/*v*) of Dulbecco’s modified eagle’s medium (DMEM) suspension supplemented with 400 UI/mL penicillin, 400 μg/mL streptomycin, 300 μg/mL gentamicin, and 2.5 μg/mL amphotericin B. After homogenization, the suspension was clarified by centrifugation at 1000× *g* for 15 min and used to infect Vero cells (BSCL86 ATCC, Istituto Zooprofilattico Sperimentale della Lombardia e dell’Emilia Romagna, Italy). In this experiment, we used cells from passages 100 to 120. Cells were seeded into 12-well plates in DMEM supplemented with a cocktail of antibiotics (final concentration 100 UI/mL of penicillin, 100 µg/mL streptomycin, and 0.5 µg/mL amphotericin B) and 10% (*v*/*v*) FBS and kept in a thermostatic water bath at 37 °C in an atmosphere of 5% CO_2_. Once the monolayer was 80–90% confluent, the cells were deprived of the culture medium and incubated with 0.5 mL of supernatant containing ORFV for 90 min at 37 °C in an atmosphere of 5% CO_2_. For each sample we used 3 wells. In 3 wells the cells were not in contact with the virus being a negative control. After incubation, the cells were washed 3 times with 1× PBS, and new culture medium with a cocktail of antibiotics (final concentration 100 UI/mL penicillin, 100 µg/mL streptomycin, and 0.5 µg/mL amphotericin B) and 2% (*v*/*v*) FBS was added. Cultures were checked daily for the presence of cytopathic effects (CPEs). When a CPE was observed or within 5 days, the cultures were freeze–thawed 3 times, harvested, and centrifuged at 200× *g* for 10 min. After centrifugation, the supernatant was collected and stored at −80 °C and was ready to be used for any subsequent analysis.

### 2.4. ORFV DNA Extraction, PCR and Sequencing

To confirm the presence of ORFV, viral DNA was extracted from 25 mg of each lesion tissue sample using the DNeasy Blood and Tissue Kit (Qiagen) according to the manufacturer’s instructions. When possible, DNA was extracted from different lesions of the same host (see Table 1 for details).

The genes of the ORFV genome, B2L (encoding the major envelope protein), O45 (encoding the late transcription factor VTLF-1), and VIR (encoding the dsRNA-binding protein), were amplified. For B2L, a semi-nested PCR, which was carried out with the PPP3-PPP4 primers (producing a 235 bp fragment) on a 570 bp-long B2L fragment, was perfomed. For O45, a specific *Parapoxvirus* fragment (size 400 bp) was amplified using the O45F and O45R primers. VIR was amplified using the VIR1 and VIR2 primers which provided fragments of 617 bp ([21,23,24], respectively). Additionally, to amplify VIR even for samples that did not provide good results for the first PCRs, a further set of primers (VIR 3 and VIR 4), which amplified a fragment of approximately 817 bp, was designed. PCR was performed as previously described by Kottaridi et al. [24], with slight changes in annealing temperatures (see Table 2 for details on primers). The location within the ORFV genome of the genes analyzed in the present study is reported in Supplementary Figure S1 (see the reference inside [25]). Positive (old high-quality ORFV DNA samples that always give good results when amplified) and negative controls were used to test the effectiveness of PCR protocols and the absence of possible inhibitors or contamination. Electrophoreses were carried out with an Invitrogen E-Gel EX 2% agarose kit (gel precast). The specific DNA PCR bands were excised from the gel and purified using a QIAquick Gel Extraction Kit (Qiagen). When aspecific bands were also present during electrophoresis, the PCR products were purified using the CleanSweep PCR Purification kit (Thermo Fisher Scientific). PCR products were sequenced for both forward and reverse strands (by means of the same primers used for PCR) using a Sanger Sequencing 3500 Series Genetic Analyzers Terminator 3.1 apparatus (Applied Biosystems).

### 2.5. Phylogenetic and Phylogeographic Analysis

Eighteen sequences were obtained for each gene in the present study; they were aligned using the Clustal Omega package [26] (available at https://www.ebi.ac.uk/Tools/msa/clustalo/, accessed on 10 April 2021) after editing by means of Unipro UGENE v.35 [27], and subsequently deposited in GenBank (see Supplementary Table S1 for accession numbers). A concatenated dataset including all 3 genes analyzed (in the following natural order: B2L, VIR, O45) was also created with the aim of analyzing a long DNA fragment showing the level of polymorphism and genetic variability to the highest degree possible.

The values of genetic variation were assessed without considering gaps, estimating the number of polymorphic sites (S) and haplotypes (H), nucleotide diversity (π), and haplotype diversity (h) using the software package DnaSP 6.12.03 [28].

To perform molecular analysis for the consideration of our data in a wider geographic context, we constructed a second dataset which included our 18 Sardinian sequences of the VIR gene, together with a sequence of *Pseudocowpox* virus (GQ329670) as an outgroup, and all the sequences corresponding to the same ORFV gene deposited in GenBank (last update October 2020). We obtained a full dataset representative of viral strains from all continents (Africa (1), Asia (93), Europe (37), North America (10), Oceania (1), and South America (19)). Only the VIR sequences from GenBank that exactly matched the VIR fragment analyzed in this study were used for the analysis.

For each dataset analyzed, the best probabilistic model of sequence evolution was determined individually by using jModeltest 2.1.1 [29], with a maximum likelihood optimized search. To verify the reliability of the dataset for phylogenetic and phylodynamic purposes, the phylogenetic signal [30] for the datasets of the VIR gene was tested by means of a likelihood mapping analysis of 10,000 random quartets using TreePuzzle software [31]. MrBayes 3.2.7 [32] was employed to perform a Bayesian phylogenetic analysis, setting nst = 3, rates = gamma, and ngammacat = 4 as model parameters. Two independent runs, each consisting of 4 Metropolis-coupled Markov chain Monte Carlo (MCMC) chains (1 cold and 3 heated chains), were run simultaneously for 5 million generations, sampling trees every 1000 generations. The first 25% of sampled trees were discarded for burn-in. Runs were carried out by means of the CIPRES Phylogenetic Portal [33]. The convergence of chains was verified by checking the average standard deviation of split frequencies (which should approach 0) [32] and the potential scale reduction factor (which should be approximately 1) [34] following Scarpa et al. [35]. Phylogenetic trees were visualized and annotated using FigTree 1.4.0 (available at http://tree.bio.ed.ac.uk/software/figtree/, accessed on 10 April 2021).

Dating was performed using a Bayesian approach under the MCMC algorithm implemented in Beast 1.10.4 software [36] by means of the collection year of the samples. To identify the best clock model for dating analyses, both strict and uncorrelated log-normal relaxed clock models were tested with fast runs of 100 million generations. Selection was performed by comparing Bayes factor values by using Tracer 1.7 [37]. Similarly, all available demographic models (both parametric and nonparametric) were tested. After the selection, for both datasets, the phylogenetic time-scaled trees and the evolutionary rates were co-estimated running 300 million generations, with sampling every 30,000 generations. The resulting log files were inspected by using Tracer 1.7 software [37]. Only values of ESS (effective sample size) ≥200 were accepted. The maximum clade credibility tree was drawn, visualized, and annotated by means of TreeAnnotator (Beast package) and FigTree, respectively.

Beast software was also used for further runs under the coalescent Bayesian skyline demographic model [38] assuming a piecewise constant model with 10 coalescent intervals, importing the previously obtained evolutionary rates with a strict clock model. The log files and time trees obtained were used to draw the Bayesian skyline plot (BSP) to explore variation in genetic diversity along the temporal scale for both worldwide and Sardinian populations.

A median-joining network [39] was constructed using the software package Network 10.0.0.0 (www.fluxus-engineering.com, accessed on 10 April 2021) to infer the genetic relationships among the Sardinian haplotypes and to detect the occurrence (if any) of discrete genetic clusters. The transitions and transversions were equally weighted. Due to the lack of knowledge regarding the possible occurrence of retromutation events, the same weight was assigned to all of the observed polymorphisms.

## 3. Results

### 3.1. Clinical Findings

Clinically, the prevalence of ORFV infection was higher in lambs and kids than in adult animals. Newborn lambs and kids showed ORFV lesions 2–3 days after birth, and most of them completely recovered within a few weeks. However, lambs with more severe lesions died at approximately 1 month of age as they were unable to feed.

**Figure 2:**
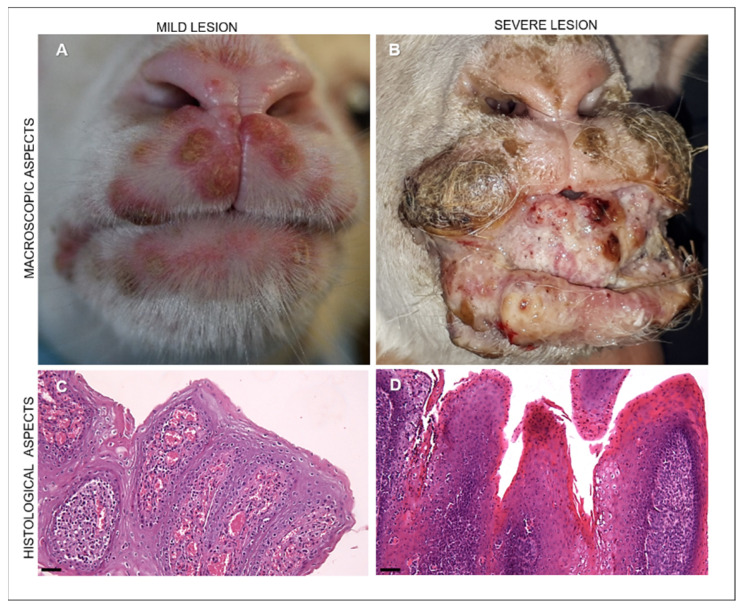
Representative images of the macroscopic appearance of ORFV-infected lambs and microscopical patterns of ORFV-infected lambs with mild and severe lesions. (**A**) Mild lesions in an ORFV-infected lamb showing vesicles and crusted pustules in the upper and lower lips. (**B**) Severe lesions appearing in an ORFV-affected lamb as coalescing hyperemic, proliferative, verrucous outgrowths with a papilloma-like appearance covering the incisor tooth, present in the lips together with crusted lesions. (**C**) Mild lesions histologically showing moderate epithelial hyperplasia, hyperkeratosis with elongated rete ridges, and proliferations of neovascular structures in the dermis of the host skin. Hematoxylin and eosin staining (H&E). Original magnification 100×. Scale bar = 100 µm. (**D**) Severe manifestation of ORFV-infected animals microscopically characterized by massive proliferative patterns involving the epithelium and showing hyperkeratosis and hyperplastic epithelium with extremely elongated rete ridges and ballooning degeneration. In the lamina propria of the buccal mucosae and in the derma of skin, there is evident proliferation of mesenchymal cells resembling hemangiomatous patterns. Hematoxylin and eosin staining (H&E). Original magnification 100×. Scale bar = 100 µm.

In young animals, lesions were usually localized in the skin of the muzzle as well as in the mucosae of the lips, gums, and tongue. Macroscopically, we observed lesions, hereafter indicated as “mild”, appearing as crusted pustules and vesicles (Figure 2A), and lesions, hereafter indicated as “severe”, appearing as coalescing hyperemic, proliferative, verrucous outgrowths with a papilloma-like appearance (Figure 2B).

In adult animals, we observed mild lesions appearing as crusted pustules in the muzzle and, rarely, small fibroproliferative lesions in the udder (data not shown).

**Figure 3:**
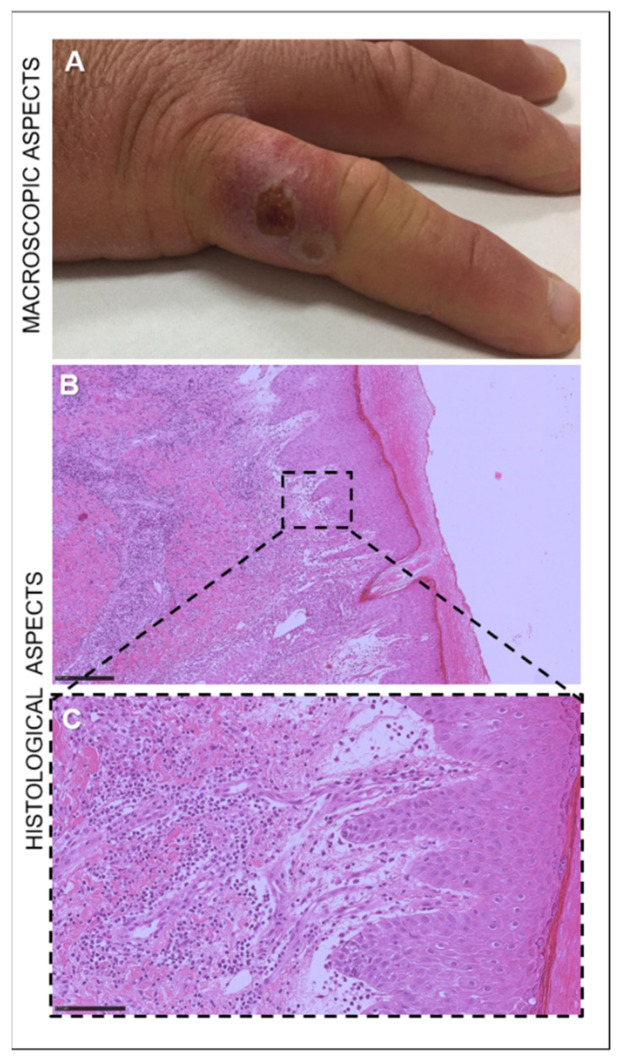
Nodule on the right little finger of a 30-year-old sheep farmer infected with ORFV. (**A**) Targetoid nodule with a necrotic center, a white ring, and peripheral erythema. (**B**) Microscopical appearance of the skin of the ORF-infected man, characterized by marked acanthosis and hyperkeratosis. Hematoxylin and eosin staining (H&E). Original magnification 50×. Scale bar = 250 µm. (**C**) In the dermis, vascular proliferation with granulocytic and eosinophilic infiltration. Hematoxylin and eosin staining (H&E). Original magnification 400×. Scale bar = 100 µm.

In the 30-year-old sheep farmer who was brought to our attention, a red plaque on a finger was observed. The lesion evolved into a targetoid nodule with a necrotic center, a white ring, and peripheral erythema (Figure 3A). The nodule was subsequently followed by a dry crust and resolved without scarring.

### 3.2. Histopathology

Microscopically, in animals with mild lesions we observed moderate epithelial hyperplasia and hyperkeratosis with extremely elongated rete ridges, ballooning degeneration, and rare eosinophilic inclusion bodies. In the lamina propria of the buccal mucosae and in the dermis of the skin, proliferation of neovascular structures was noted (Figure 2C). The severe manifestation of ORFV infection was histologically characterized by massive proliferative patterns involving the epithelium and the fibrovascular component of the lamina propria of the mucosae as well as of the dermis of the skin (Figure 2D). Marked acanthosis, hyperkeratosis with parakeratotic crusts, and edema of the superficial dermis were histopathologically observed in the human lesion (Figure 3B). In addition, the dermis exhibited vascular proliferation together with granulocytic and eosinophilic infiltration, showing a tendency towards abscessualization (Figure 3B,C).

### 3.3. Virus Isolation

**Figure 4:**
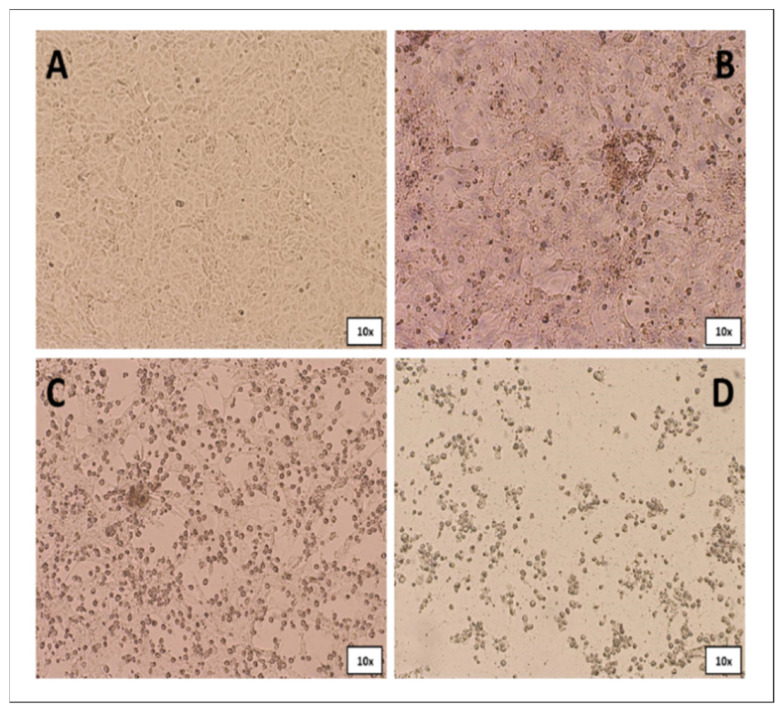
ORFV CPE resulting from infection by scab homogenate on VERO cells. (**A**) mock; (**B**) early stage; (**C**) intermediate state; (**D**) final stage.

ORFV was isolated from VERO cells in 14 out of the 18 specimens examined (5 kids, 3 ewes, and 6 lambs). A cytopathic effect was detected on the confluent monolayer approximately 2 days after infection, including rounding, ballooning, degeneration, and detachment (Figure 4).

### 3.4. Molecular Virus Identification

After sequencing, all samples resulted positive for the presence of ORFV.

The DNA needed to perform sequencing reactions was extracted from viral isolates for the 14 samples that we were able to isolate. When it was impossible to isolate the virus (4 samples, 3–4–5–18 in Table 1), the DNA previously extracted from lesion tissues to confirm the presence of ORFV was also used for sequencing.

### 3.5. Phylogenetic and Phylogeographic Inferences

The sequences obtained for the B2L, O45, and VIR genes during molecular virus identification were also used to perform phylogenetic and phylogeographic analyses. The GenBank accession numbers of the sequences that we obtained are reported in Supplementary Table S1.

Among the 3 genes sequenced here, we obtained the largest fragment for the VIR gene and the smallest fragment for the B2L gene. Overall, the highest levels of genetic diversity were found for the VIR gene (see Table 3 for genetic divergence estimates).

A 933 bp-long concatenated (B2L-VIR-O45) gene dataset for the 18 Sardinian samples was obtained to perform phylogenetic and network analyses on the Sardinian ORFV strains. The size of the fragments for each gene used to obtain this merged dataset corresponded to the portion of sequence that was scorable and more easily overlapped with the other fragments. Furthermore, the size of the B2L-VIR-O45 concatenated fragment was smaller than expected considering the size of the gene concatenated, as a consequence of the use of a shorter reference sequence (GQ329670) during dataset assembly. This dataset showed 49 polymorphic sites that defined 13 haplotypes (see Table 3 for details on genetic divergence estimates). 

**Figure 5:**
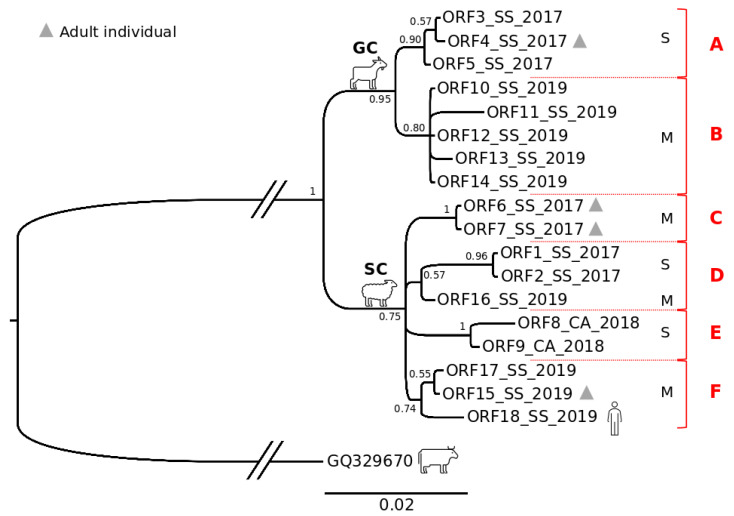
Bayesian phylogenetic tree based on the Sardinian ORFV concatenated gene dataset (B2L-VIR-O45). Values of node supports are expressed in posterior probabilities. The sample codes are as reported in Table 1. S: severe pattern of lesions; M: mild pattern of lesions. Red capital letters on the right indicate the genetic sub-groups described in the text.

The tree obtained for this concatenated dataset (Figure 5) also included a concatenated gene sequence of the *Pseudocowpox* virus (GQ329670) as an outgroup and evidenced a well-supported structuring between the groups of sequences isolated from sheep and goats. Within these two monophyletic sister clusters (SC and GC for sheep and goats, respectively), an internal, also monophyletic, substructure was found, generally consistent with the severity (mild vs. severe) of the anatomopathological changes observed in the hosts. In particular, two internal subgroups (Group A and Group B) were retrieved for the GC cluster. Group A included viral strains isolated in 2017 on a farm located in the northeast of the island (samples 3–5 in Figure 1) from one adult goat and two kids all showing severe ORFV-related lesions, while Group B included viral strains isolated in 2019 (samples 10–14 in Figure 1) from kids with mild lesions on a farm which was 105 km away in the northwest of the island. In the SC cluster, 4 internal subgroups were found (Group C, Group D, Group E, and Group F). Group C included viral strains isolated in 2017 (samples 6 and 7 in Figure 1) from ewes with a mild pattern of lesions on one farm in the northwest of the island. Group D included viral strains isolated from lambs in the same area of the island in 2017 (samples 1 and 2 in Figure 1) and 2019 (sample 16 in Figure 1). In particular, the viral strains isolated in 2017 belonged to animals living on the same farm with severe patterns of lesions, while the viral strain isolated in 2019 belonged to a lamb living on a different farm 45 km away, with a mild pattern of lesions. Group E included viral strains isolated in 2018 (samples 8 and 9 in Figure 1) from lambs of one farm in the south of the island, which showed a severe pattern of lesions. Group F included viral strains isolated in 2019 (samples 15 and 17 in Figure 1) in one ewe and one lamb from two farms approximately 90 km apart in the northwest and northeast of the island, respectively. All animals showed a mild pattern of lesions. This latter group also included a viral strain isolated in 2019 (sample 18 in Figure 1) in the north of the island from a lesioned skin sample of a farmer. Interestingly, each main cluster in the phylogenetic tree is monophyletic and includes viral strains isolated in the same year (with only one exception for a sequence in Group D). No relevant structuring based on the geographic distribution of hosts was found among viral strains.

**Figure 6:**
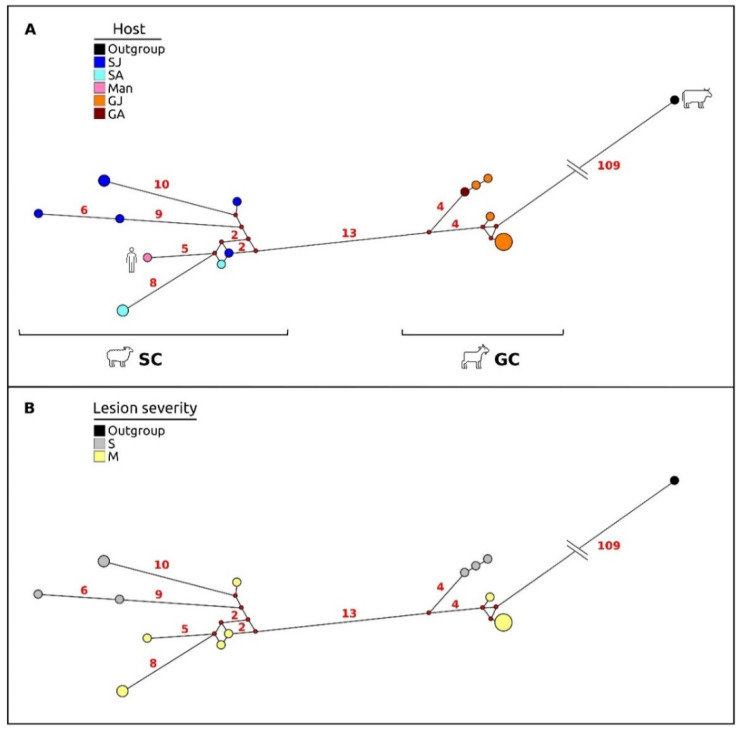
Median-joining network based on the Sardinian ORFV concatenated dataset (B2L-VIR-O45). (**A**) The haplotype spots in the graphic are colored according to host species (sheep vs. goats). SJ: lamb; SA: ewe; GJ: kid; GA: goat. (**B**) The haplotype spots in the graphic are colored according to the type of lesions showed by hosts. S: severe pattern of lesion; M: mild pattern of lesion. The small red plots on the nodes show median vectors representing the hypothetical connecting sequences that were calculated using the maximum parsimony method. Only the numbers of mutations between haplotypes that are greater than 1 are reported on network branches.

In keeping with these results, the network analysis (Figure 6A) showed a high level of variation among viral sequences, with the occurrence of two highly divergent clusters (SC and GC), which were consistent with those retrieved by the phylogenetic tree analysis, and exclusive to sheep (SC) and goats (GC). These two clusters were separated by 13 point mutations occurring in the central portion of the VIR gene fragment analyzed, which accounted for 12 amino acid changes, 9 of which involved different classes of amino acids. Goat viral strains grouped within the GC cluster, were the closest to the outgroup (*Pseudocowpox* virus). When considering the network (Figure 6B) in accordance with the clinical presentation of the infected animals, the results showed that viral strains isolated from severe lesions diverged significantly from those associated with mild lesions in both sheep and goats.

In particular, within the SC cluster the group of viral haplotypes isolated from severe lesions diverged from those isolated from mild lesions as a consequence of mutations occurring mainly at the VIR gene, while within the GC cluster, the mutations that differentiated the group of haplotypes isolated from severe lesions from those isolated from mild lesions equally involved the three genes analyzed (B2L, VIR, O45).

**Figure 7:**
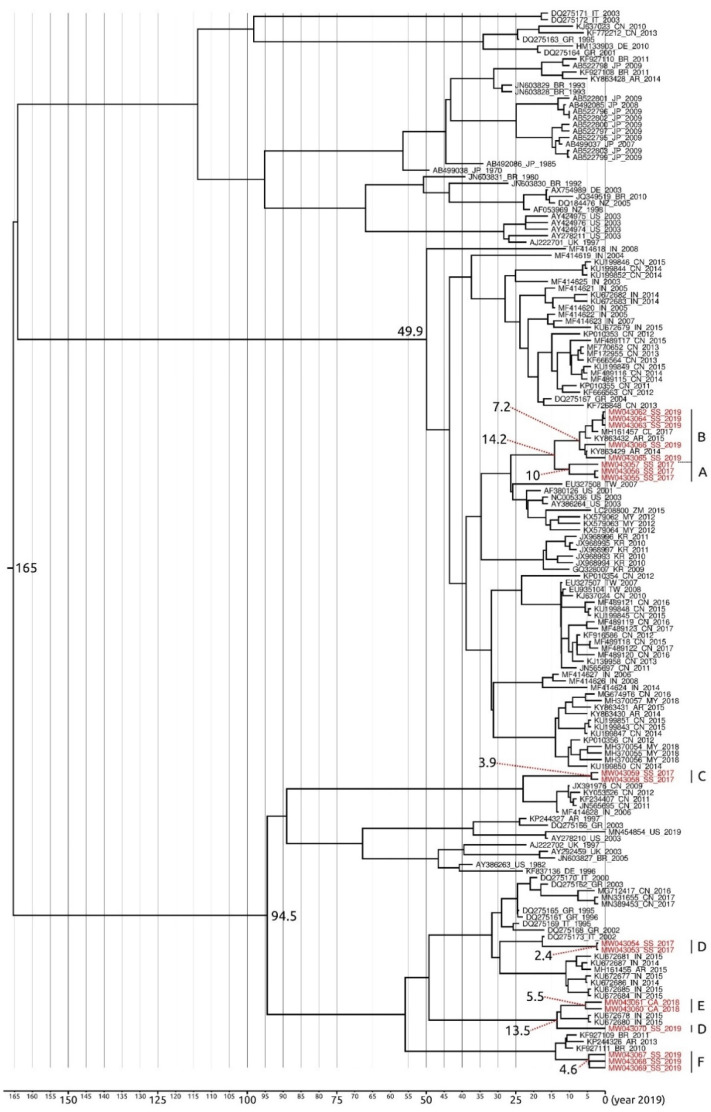
Bayesian phylogenetic time-scaled maximum clade credibility tree based on the ORFV VIR global dataset. All main nodes of the tree are highly supported by values of posterior probabilities ≥0.95. Nodes with a percentage of posterior probability <0.95 were considered to be not statistically supported. The Sardinian samples codes are reported in red font (further details in the supplementary Table S1). The codes of sequences from GenBank report the accession number, the country acronym, and the presumptive year of isolation. The numbers at the nodes refer to the dating (in years before 2019) of the cluster. Capital letters on the right refer to the genetic sub-groups indicated in Figure 5. Horizontal bar indicates the years before 2019 in a temporal scale.

A 383 bp-long dataset that grouped the Sardinian sequences of the VIR gene obtained in the present study along with its relatives outside the island (144) from GenBank (see Figure 7 for countries and GenBank accession numbers) was created to perform a second phylogenetic tree analysis.

The likelihood map (Supplementary Figure S2a [30,31]) calculated using this dataset evidenced a strong phylogenetic signal according to Schmidt et al. [31], with 2.8% of dots falling in the central area of the triangles, indicating that the dataset could be considered biologically reliable for phylogenetic inference [30].

Seventy-nine VIR haplotypes (not considering gaps) were found (see Table 3 for genetic divergence estimates); in particular, all the Sardinian haplotypes isolated from sheep, along with those isolated in 2017 from goats, were private to the island and had never been reported before, while haplotypes isolated from Sardinian goats in 2019 corresponded to a viral sequence already isolated in 2017 in occupationally exposed women in Chile (GenBank # MH161457) and in 2014 in goats from five outbreaks in Argentina (GenBank # KY863429).

Phylogenetic analyses performed by means of Beast and MrBayes softwares generated trees with the same general topology. We therefore reported only the Bayesian phylogenetic time-scaled maximum clade credibility tree generated by Beast software (Figure 7).

Overall, our phylogenetic tree analysis indicated that the Sardinian VIR strains isolated in goats lay within a well-supported cluster, also grouping sequences from Chile and Argentina, that diverged from its most recent common ancestor (MRCA) 14.2 years before 2019. This cluster belongs to a larger clade, whose radiation interested almost exclusively strains infecting goats, which occurred 49.9 years before 2019. Interestingly, the Sardinian strains isolated in 2017 in goats with a severe pattern of lesions differentiated approximately 10 years before 2019, while the common ancestor of the clade that includes Sardinian strains isolated in 2019 in goats with a mild pattern of lesions differentiated approximately 7.2 years before 2019.

All the VIR strains isolated from Sardinian sheep, along with the one isolated from the farmer, are included in a large clade whose radiation occurred 94.5 years before 2019. In particular: (i) the strains isolated from ewes with mild lesions in 2017 differentiated 3.9 years before 2019; (ii) the viral strains isolated in lambs with severe lesions in 2017 date back to 2.4 years before 2019; (iii) the strains isolated in lambs with severe lesions in 2018 differentiated approximately 5.5 years before 2019 (however, they share a MRCA with one strain isolated from one ewe with mild lesions in 2019 which dates back to 13.5 years before 2019); and (iv) the strains isolated from an ewe, a lamb, and a man with mild lesions in 2019 differentiated 4.6 years before 2019.

In general, the time to the most recent common ancestor (TMRCA) of the large worldwide-diffused phylogenetic clade, which includes the whole Sardinian sample of VIR strains isolated from both goats and sheep, dates back to 165 years before 2019. In particular, the older Sardinian VIR strain differentiated in goats with mild lesions approximately 10 years before 2019, while the most recent strain originated in sheep with severe lesions 2.4 years before 2019.

**Figure 8:**
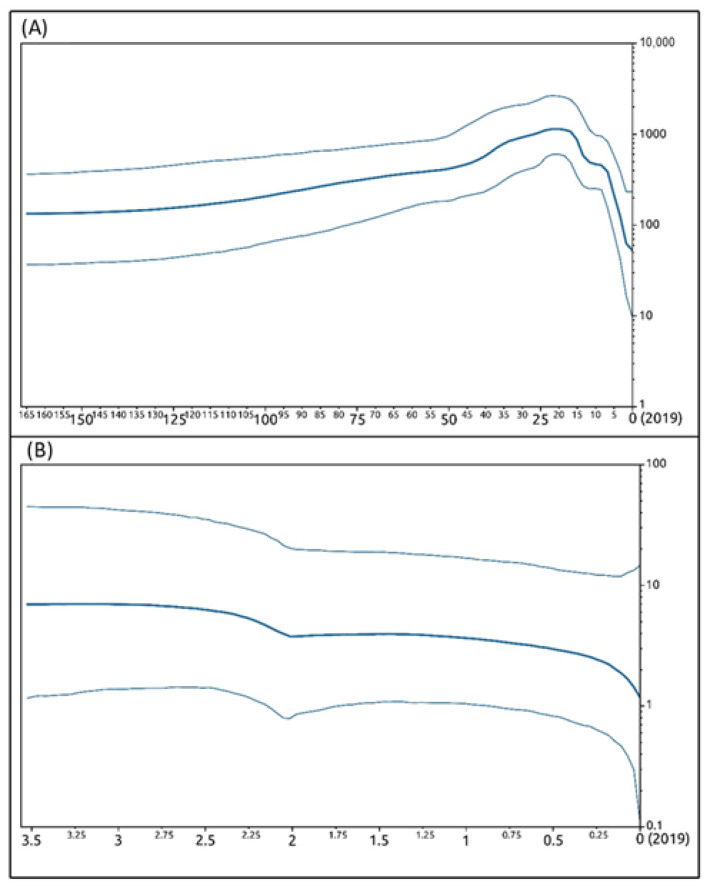
Bayesian skyline plots. Bayesian skyline plots depicting the evolutionary history of ORFV VIR gene. The effective population size of viral infection (*y*-axis) is shown as a function of time (*x*-axis), with the units expressed as years. (**A**) VIR global dataset; (**B**) VIR regional dataset encompassing only Sardinian sequences. The bold lines denote the median effective number of infections and the thin lines (placed above and below the median value line) demarcate the 95% highest posterior density (HPD) confidence interval.

Under the coalescent constant size (CCS) lognormal uncorrelated relaxed clock model, the viral evolutionary mean rate calculated for the whole VIR gene dataset was estimated to be 8.18 × 10^−3^ substitution/site/year, with a 95% HPD of 1.10 × 10^−3^–1.70 × 10^−2^. Patterns of viral spreading inferred by the BSP (Figure 8A) showed a general trend with an initial long-lasting constant size expansion of the global viral population among hosts which started when the ancestor of the ORFV VIR gene differentiated (approximately 165 years before 2019) and was followed by a decrease in viral spread that began about 20 years before 2019. On the other hand, the analysis also evidenced (Supplementary Figure S3a) that from the first radiation of the virus, the amount of its lineages distributed worldwide constantly increased, with a general exponential growth up to the present day.

The viral evolutionary mean rate was also calculated for the Sardinian VIR gene dataset, including the 18 sequences obtained in the present study. Its value was estimated to be 5.33 × 10^−4^ substitution/site/year with a 95% HPD of 3.06 × 10^−4^–8.10 × 10^−4^. The patterns of demographic history inferred by the BSP (Figure 8B) showed a general constant size expansion for the ORFV population among hosts during the whole sampling campaign (2017–2019), with a low decrease in viral spread during the final part of 2017.

The likelihood map (Supplementary Figure S2b [30,31]) calculated on this Sardinian VIR gene dataset showed a strong phylogenetic signal [31], with 5.8% of dots falling in the central area of the triangles, thus indicating in this case also that this dataset can be considered biologically reliable for phylogenetic inference [30].

The amount of VIR lineages in Sardinia (supplementary Figure S3b) was constant during the period considered in the present study, with a slight increase likely before the first years of the sampling campaign (2–3.5 years before 2019), which was followed by a plateau reached in 2017 that maintained the amount of viral lineages constant on the island, at least up to 2019.

## 4. Discussion

To the best of our knowledge, this study represents the first indicative report of the presence of ORFV on a Mediterranean island with a very large sheep and goat population in which the evolution of the virus has been evaluated and described from a molecular point of view.

By means of three molecular markers used to investigate the evolutionary and phylogeographic patterns of ORFV in Sardinia, we found that this virus shows a generally high level of genetic variability in the world, with a mutational evolutionary rate at the VIR gene that is an order of magnitude higher than that detected for the viral strains isolated in Sardinia. Such a finding is in accordance with the global constant expansion of ORFV allelic variants (lineages) reported in the present study. The rise of several new viral lineages outside the island is a possible consequence of the occurrence of a generally diffused selective pressure that may have prompted worldwide high evolutionary rates and new allelic variants for this virus. This evolutionary trend was also reported for a number of other taxa [40,41,42]. The global evolutionary trend detected for ORFV in the present study reflects an initial constant size spread of the viral population starting from the moment in which the ancestor of the VIR gene differentiated (approximately 166 years ago) until the beginning of the 2000s. Indeed, in the 2000s, a general decrease in the virus worldwide diffusion was evidenced to be likely connected with the decline in the number of sheep reported between 1992 and 2018 for the Americas (−19%), Europe (−41%), and Oceania (−22%), which resulted in a total loss of approximately 70 million animals (source: Food and Agriculture Organization of the United Nations, 2020—http://www.fao.org/faostat/en/?#data/, accessed on 10 April 2021). During the same period, a relevant decrease in the number of goats was also reported for the Americas (−6%) and Europe (−28%), resulting in a loss of more than 4 million heads (source: Food and Agriculture Organization of the United Nations, 2020—http://www.fao.org/faostat/en/?#data/, accessed on 10 April 2021).

However, in Sardinia, consistent with the lower evolutionary rate that we found for ORFV, the variation in the amount of viral lineages increased slightly until 2017, thus contracting their expansion in the following years. Such a result, even if obtained on a restricted temporal scale, likely reflects the evolutive trend that generally involves allelic variants at the end of processes of adaptation to new habitats [43], suggesting that the Sardinian ORFV population might have reached equilibrium in 2017.

Thus, in Sardinia, our results also evidenced a general constant size spread of the ORFV population among hosts during the sampling period (2017–2019). The only exception was observed for the last months of 2017, when a slight decrease in viral expansion occurred as a possible consequence of the temporary contraction of the effective size of the host population that was also reported by local authorities. Indeed, according to the BDN of the Zootechnical Register established by the Italian Ministry of Health at the CSN of the “G. Caporale” Institute of Teramo (Italy), the northern province of Sassari, which accounts for 89% of the viral strains analyzed in the present study, was the only area of the island where the number of sheep and goats decreased over the period 2016–2019. In particular, from 2016 to 2018, in this province the number of sheep decreased by 9.5%, and the number of goats decreased by 14%, resulting in a statistically significant general loss of 55,978 sheep and 5344 goats for the whole island (a chi-squared test was performed in the present study to evidence the significance of host numbers decreasing, data not shown).

The Sardinian ORFV strains found in the present study originated between 2009 and 2016/2017, and the viral haplotypes are almost all exclusive to the island. The only exception was represented by a goat strain isolated in 2019, which showed a VIR sequence identical to those already reported in 2014 and 2017 for humans and goats in South America. Such a finding suggests that this ORFV haplotype, which dates back to 2012, likely originated outside the island in an area of the world that is probably a center of animal trade involving both the Mediterranean and southern America. In such a context, the Iberian Peninsula could represent the trade union for goat exchanges between the two continents [44,45,46].

However, we cannot exclude the possibility that a phenomenon of evolutionary convergence may be invoked for the occurrence of identical ORFV VIR haplotypes in the Mediterranean and South American regions as a possible consequence of similar environmental conditions and selective pressure acting on hosts and viruses. Indeed, these countries share either comparable conditions, with a warm and humid climate suitable for the spread of the virus, or high concentrations of livestock [12,47,48].

Regarding the genetic clusters that are private to Sardinia, the most recent cluster originated in 2016/2017 among sheep in the north of the island, while the oldest also originated in the north of the island, but in 2009 among goats. These findings suggest a high plasticity of ORFV in Sardinia that is consistent with the value of evolutionary rate that we found for the virus on this Mediterranean island. Indeed, the mutational rate found for the Sardinian strains seems to be high enough to allow ORFV to adapt quickly and genetically differentiate in each microarea where it spread before reaching an equilibrium in 2017; thus resulting in several genetic groups that differ, not only as a consequence of the hosts infected, but also as a possible result of the environmental conditions present on each farm. A similar trend was also found for the rabies virus in Cuba, where variants showed some degree of regional structuring independent of the host involved [49]. In this context, in Sardinia peculiar biological factors, that should be evaluated deeply in the future, may have further prompted the differentiation between ORFV lineages associated with severe and mild lesions in hosts.

Regarding the genetic structuring among ORFV variants, as already reported for many geographic areas of the world [16], a well-supported genetic structuring due to host-specific nucleotide substitutions was also found in Sardinia between the viral strains isolated from sheep and goats. The high genetic divergence of ORFV strains between the two hosts may explain the failure of the ORFV vaccine developed for sheep when applied to goats [22]. In particular, such a failure could be related mainly to the divergence occurring between the viral sequences encoding neutralizing epitopes in sheep and goats [22]. Using a combination of different strains of the virus of the two species in vaccine preparations could make it possible to overcome the problem of the different immune responses that they cause [12]. Seven to 10 days after vaccination, the immune response of the host to ORFV is triggered by a significant response by neutralizing antibodies, and it remains active for years, with the possibility of being strengthened by vaccination boosters [12]. Furthermore, another important role in the immune response is carried out by cell-mediated immunity (CMI), and it seems that the CMI occurs before the production of neutralizing antibodies [5,12]. In the light of this consideration, vaccination is to be considered the most important tool in the fight against ORFV.

In the present study, we revealed 2 macroscopically and histologically distinct patterns of lesions for the ORFV Sardinian outbreaks investigated. In small ruminants, ORFV typically has a cyclic course in herds, and mild pustular/crusted lesions on the muzzle are frequently found and easily associated with virus infection. In contrast, a severe proliferative pattern is relatively rare [50], making the diagnostic process more complex. In these cases, differential diagnosis should include papillomatosis, neoplasia, ulcerative dermatosis, and chorioptic mange. Histological and virological investigations are essential to make a correct diagnosis, which is important considering the zoonotic potential of ORFV [51].

In Sardinian sheep, the viral allelic variants associated with severe lesions sequenced here differed from the variants associated with mild lesions due to the occurrence of mutations located mainly at the central portion of the VIR gene. On the contrary, in Sardinian goats, the viral allelic variants associated with severe lesions differed from those isolated from mild lesions due to mutations that were scattered homogeneously in all the three genes studied here. Such a discrepancy in the distribution of polymorphic sites throughout gene fragments suggests that the occurrence in hosts of severe lesions after ORFV infection is not necessarily linked to the expression of one of the three genes analyzed here. The type of lesions in sheep and goats could rather be the effect of the expression of another gene or represent a multifactorial character whose the manifestation is the product of the combined action of several genes. Indeed, a number of studies have focused on the analysis of pathogenic factors involved in viral immunomodulation, thus evidencing the possible connection with the expression of other genes [52,53,54,55,56,57].

Among the virulence factors identified to date, the viral vascular endothelial growth factor (VEGF) gene plays an important role in the pathogenesis and virulence of ORFV [57]. In the underlying dermis, ORFV encodes VEGF-E and promotes the angiogenesis and proliferation of epidermal cells [57]. In such a context, excessive proliferation of epithelial cells and new vascular structure growth in the lamina propria and in the dermis may also be supported by a different modulation of VEGF expression that might account for the genetic differentiation between different patterns of lesions produced by ORFV infection.

Further researches should help to uncover the pathogenetic mechanisms and the genes able to trigger a change from the classic pattern of self-limiting ORFV mild lesions (usually in a few weeks [58]) to the hemangiomatous pattern histologically determined in severe lesions.

However, the possible contribution of the interferon resistance gene (VIR) in modulating the severity of the clinical manifestation of the disease in hosts should not be excluded. Indeed, this gene, which is located in the left terminus of the ORFV genome [59] and encodes a dsRNA-binding protein that inhibits the antiviral activity of interferons in hosts, plays a role in the immunomodulation of the immune response, making its analysis fundamental to understanding the control of resistance to interferon type I operated by ORFV. This mechanism is regulated by this gene and is used to hinder host defenses against the virus. In general, modifications occurring in immunomodulating genes, regardless of their effect on virus activity, can evade the immune response of the host, and for this reason, a deep insight into the nucleotide changes of different viral strains would allow to better understand the behavior of the virus and the response of the animals to infection.

## 5. Conclusions

We investigated for the first time the evolutionary history of ORFV on the Mediterranean island of Sardinia. Furthermore, the evolutionary rate of this virus on a global scale was also calculated for the first time, thus providing new hints regarding its worldwide evolutionary history and evidencing, for the Sardinian isolates, mutational rates lower than those found for the outside areas of the world. However, the evolutionary rate of ORFV in Sardinia likely accounts for the unusual high plasticity of the virus, which might have allowed it to quickly adapt to new habitats, thus originating private strains on the island during the process of adaptation. In the future, an extensive whole genome-based approach, combined with phylodynamic analyses devoted to inferring the linkage between ORFV genetic variation and biological parameters, would help us to shed light, not only on the genetic differentiation between the ORFV strains isolated from sheep and goats, but also on the candidate genes that are involved in the host immunity response to viral infection and on the expression of specific patterns of lesions in hosts.

## Figures and Tables

**Table 1:** Sampling plan. The table reports data on the sampling collection. The sample code corresponds to the codes used for the map in Figure 1.

Code	Host	Age	Site and Type of Lesions	Sampling Year	Municipality
1	Sheep	Lamb	Muzzle and gum (proliferative lesions)	2017	Putifigari
2				2017	
3	Goat	Kid	Muzzle (proliferative lesion)	2017	Arzachena
4			Lip (proliferative lesion)	2017	
5		Adult	Udder (fibroproliferative lesion)	2017	
6	Sheep	Adult	Lip (crusted pustules)	2017	Sassari
7				2017	
8		Lamb	Limb	2018	Cagliari
			Eye		
			Ear		
			Lip		
			Nose		
			Submandibular lymph nodes		
			(proliferative lesions)		
9			Tail	2018	
			Submandibular lymph node		
			(proliferative lesions)		
10	Goat	Kid	Gum (crusted pustules)	2019	Mores
11				2019	
12				2019	
13			Lip (crusts)	2019	
14			Tongue (crusted pustules), lower lip (crust), gum (crusted pustules)	2019	
15	Sheep	Adult	Crusts	2019	Campanedda
16		Lamb	Muzzle and paraorbital region (crusts)	2019	Palmadula
17			Lip (crusts)	2019	Tempio Pausania
18	Man	Adult	Hand (proliferative lesion)	2019	Sassari

**Table 2:** List of the primers used in the present study. The table reports the sequences of the primers used to amplify and sequence the 3 ORFV genes analyzed. The forward PPP3 primer was coupled with the reverse PPP4 primer to perform internal nested PCRs and amplify a 235 bp-long fragment of the B2L gene.

Gene	Primer	Strand	Sequence	Size (bp)	Ta (°C)	Reference
B2L	PPP1	Forward	5′-gtcgtccacgatgcagct-3′	570	55	[23]
	PPP4	Reverse	5′-tacgtgggaagcgcctcgct-3′			
	PPP3	Forward	5′-gcgagtccgagaagaatacg-3′	235		
O45	045 F	Forward	5′-cctacttctcggagttcagc-3′	400	47	[24]
	045R	Reverse	5′-gcagcacttctcctcgtag-3′			
VIR	VIR1	Forward	5′-acaatggcctgcgagtg-3′	617	55	[21]
	VIR2	Reverse	5′-ttagaactgatgccgcag-3′			
	VIR 3	Forward	5′-tgatcaagtcgcctgca-3′	817	56	Present study
	VIR 4	Reverse	5′-acaaatctcttgagcagct-3′			

**Table 3:** Indices of genetic variation for the 3 ORFV genes sequenced for 18 Sardinian samples in the present study. N: sample sizes; bp: fragment size; S: number of polymorphic sites; H: number of haplotypes; hd: haplotype diversity; π: nucleotide diversity

	N	bp	S	H	hd	π
VIR	18	431	45	13	0.948 ± 0.039	0.03797
B2L	18	215	6	7	0.837 ± 0.057	0.00945
O45	18	336	6	6	0.810 ± 0.057	0.00550
B2L-VIR-O45	18	933	49	13	0.948 ± 0.039	0.01828
VIR global dataset	162	382	76	79	0.978 ± 0.004	0.04005

## Data Availability

The sequences of ORFV genes obtained during the present study are openly available in the GenBank nucleotide sequence database. Accession numbers: B2L-MW043017-34; VIR-MW043053-70; O45-MW043053-52.

## Supplementary Materials

The following are available online at https://www.mdpi.com/article/10.3390/life11050416/s1, Figure S1: Genomic structure of ORF virus (NC_005336) and position of the genes analyzed. Figure S2: Likelihood map for the ORFV VIR dataset, Figure S3: Increasing of viral lineages for ORFV VIR dataset, Table S1: GenBank accession numbers.
