# Supplementary material for: Molecular Insights into the Genetic Variability of ORF Virus in a Mediterranean Region (Sardinia, Italy)
Source: Life (Basel). 2021 May 4;11(5):416. doi: 10.3390/life11050416 (PMC8147818; doi:10.3390/life11050416)
Supplement: Supplementary file 1 [file life-11-00416-s001.zip › English revision certificate.pdf]

Editing Certificate

This document certifies that the manuscript

Molecular insights into the genetic variability of ORF virus in a Mediterranean region (Sardinia, Italy)

prepared by the authors

E. Coradduzza, D. Sanna, A.M Rocchigiani, D. Pintus, F. Scarpa, R. Scivoli, R....

was edited for proper English language, grammar, punctuation, spelling, and overall style by one or more of the highly qualified native English speaking editors at AJE.

This certificate was issued on **April 21, 2021** and may be verified on the AJE website using the verification code **D84B-35F3-CC64-F775-AD0F**.

Neither the research content nor the authors' intentions were altered in any way during the editing process. Documents receiving this certification should be English-ready for publication; however, the author has the ability to accept or reject our suggestions and changes. To verify the final AJE edited version, please visit our verification page at aje.com/certificate. If you have any questions or concerns about this edited document, please contact AJE at support@aje.com.

AJE provides a range of editing, translation, and manuscript services for researchers and publishers around the world. For more information about our company, services, and partner discounts, please visit aje.com.
