# Supplementary material for: Molecular Insights into the Genetic Variability of ORF Virus in a Mediterranean Region (Sardinia, Italy)
Source: Life (Basel). 2021 May 4;11(5):416. doi: 10.3390/life11050416 (PMC8147818; doi:10.3390/life11050416)
Supplement: Supplementary file 1 [file life-11-00416-s001.zip › Figure_S1.pdf]

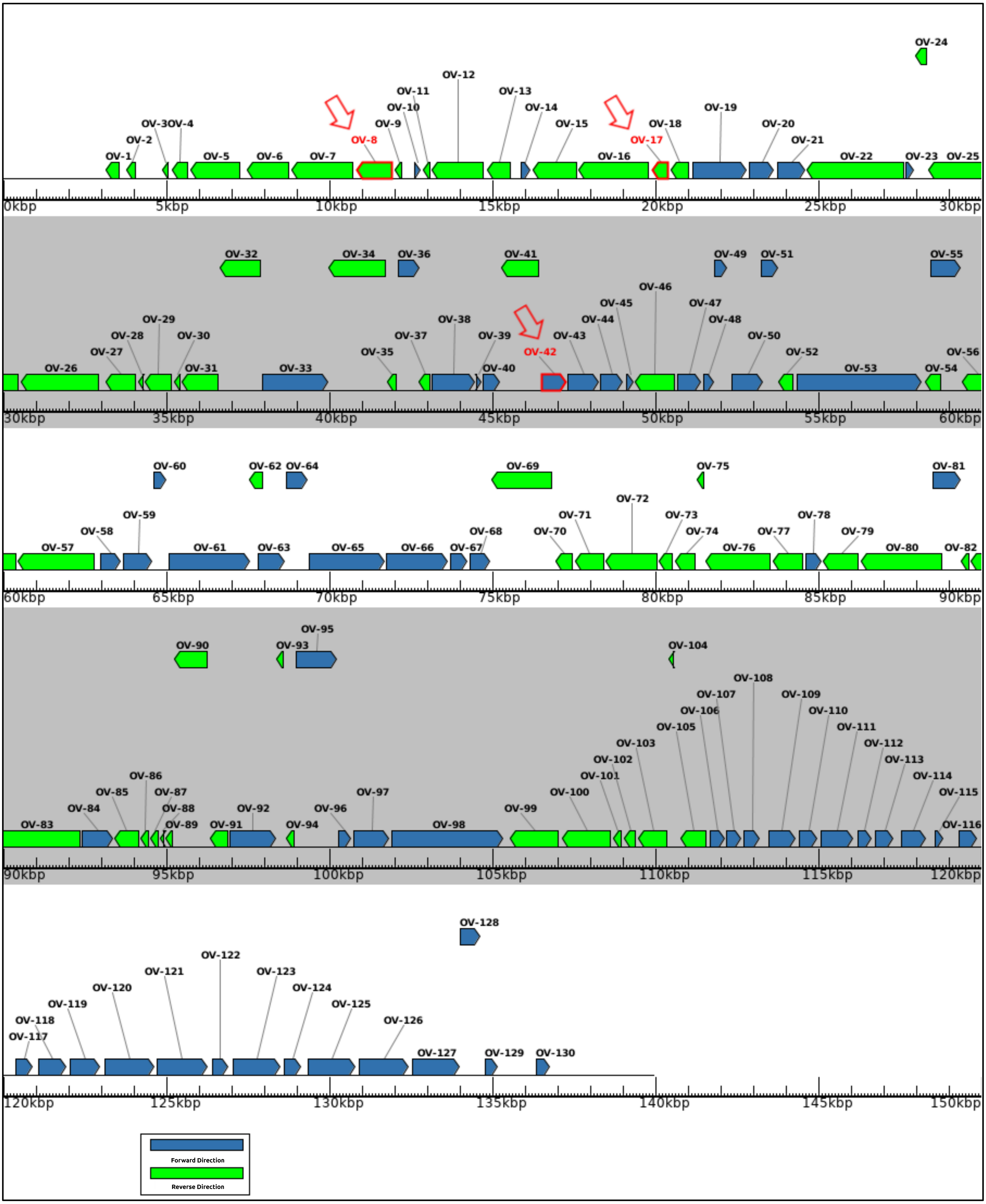


**Figure S1.** Genomic structure of ORF virus (NC_005336) sequenced by Delhon et al. [25]. Image has been obtained by means of the Genome Image Map tool implemented in ViPR (Virus Pathogen Resources, available at https://www.viprbrc.org/) and subsequently edited using the software GIMP (available at https://www.gimp.org).
The genes here analysed are outlined in red font and indicated by a red arrow. The gene B2L (ORFV011), which encodes for the major envelope protein, is indicated as OV-8, VIR (ORFV020), encoding for dsRNA-binding protein is indicated as OV-17, and O45 (ORFV045), encoding for late transcription factor VLTF-1, is indicated as OV-42.
