# Supplementary material for: Molecular Insights into the Genetic Variability of ORF Virus in a Mediterranean Region (Sardinia, Italy)
Source: Life (Basel). 2021 May 4;11(5):416. doi: 10.3390/life11050416 (PMC8147818; doi:10.3390/life11050416)
Supplement: Supplementary file 1 [file life-11-00416-s001.zip › Figure_S2.pdf]

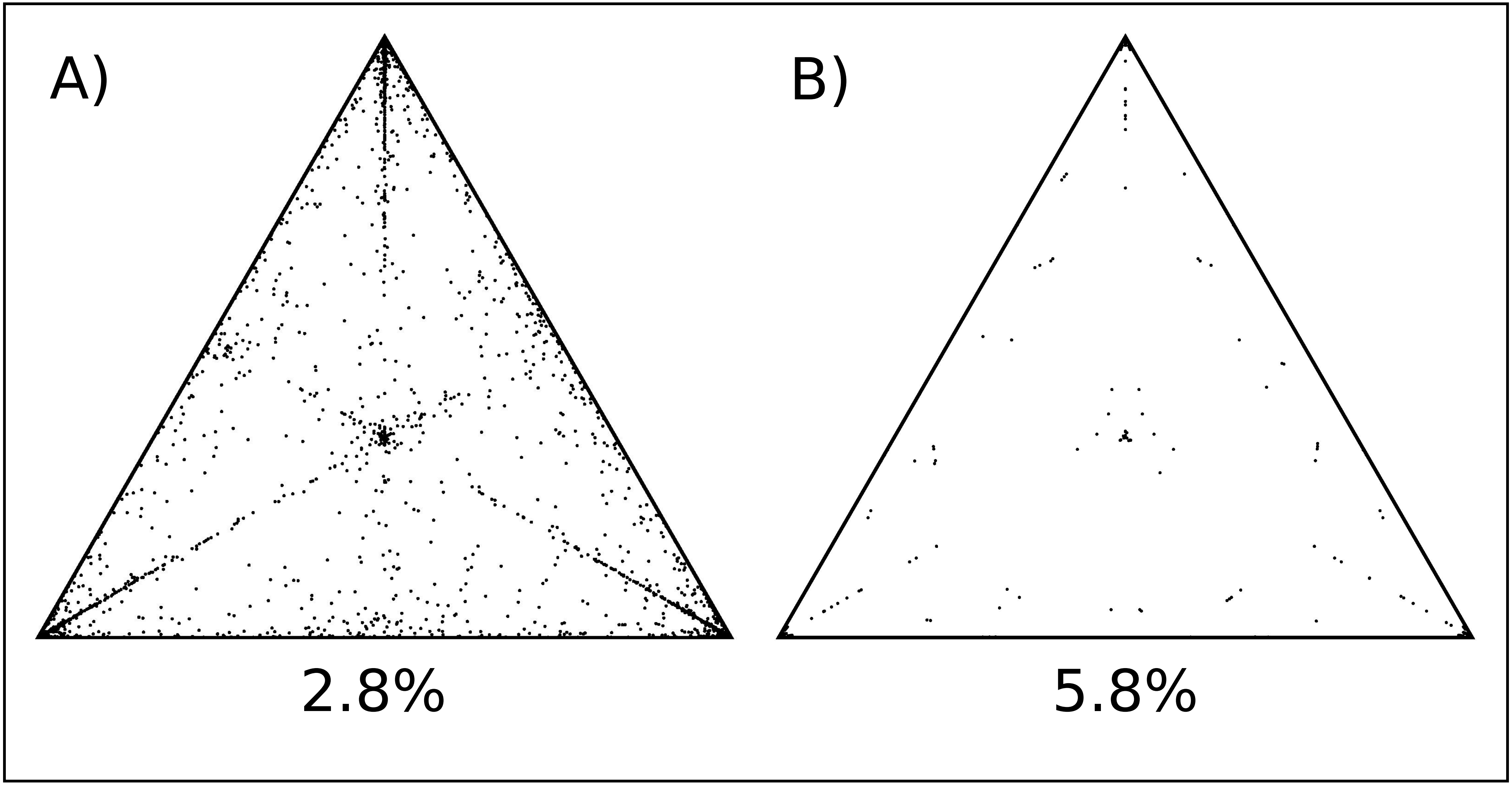

Figure S2. Likelihood map for the ORFV VIR global dataset. Likelihood map for the VIR global (A) and for the Sardinian VIR (B) datasets depicting the distribution of dots P, where P represents the likelihoods of the three possible unrooted trees for a set of four sequences [30-31]. Central area represents a star-like signal (phylogenetic noise). The value above the triangle represents the percentage of dots in the network-like area. A percentage lower than 20-30% indicates that the dataset is reliable for phylogenetic and taxonomic analyses with a good phylogenetic signal [30].
