# Supplementary material for: Molecular Insights into the Genetic Variability of ORF Virus in a Mediterranean Region (Sardinia, Italy)
Source: Life (Basel). 2021 May 4;11(5):416. doi: 10.3390/life11050416 (PMC8147818; doi:10.3390/life11050416)
Supplement: Supplementary file 1 [file life-11-00416-s001.zip › Figure_S3.pdf]

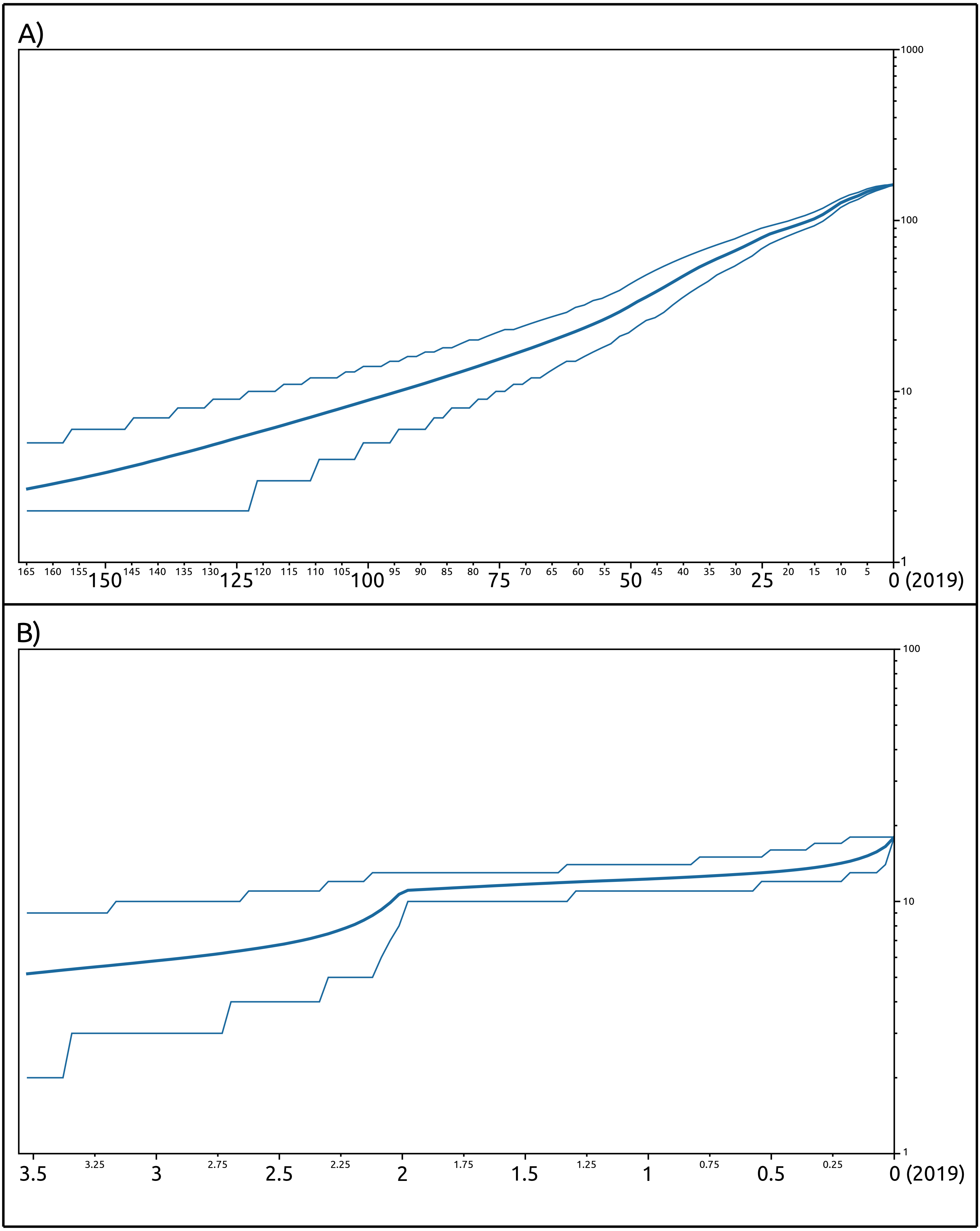

Figure S3. Increasing of viral lineages. Evolutionary increasing of VIR lineages. Logarithmic expansion of viral lineages (y-axis) is shown as a function of time (x-axis), with the units expressed as years. A) VIR global dataset; B) regional dataset encompassing only Sardinian sequences. The bold lines denote the median lineages estimates and the thin lines (placed above and below the median value line) demarcate the 95% highest posterior density (HPD) confidence interval.
