# Supplementary material for: Molecular Insights into the Genetic Variability of ORF Virus in a Mediterranean Region (Sardinia, Italy)
Source: Life (Basel). 2021 May 4;11(5):416. doi: 10.3390/life11050416 (PMC8147818; doi:10.3390/life11050416)
Supplement: Supplementary file 1 [file life-11-00416-s001.zip › Table_S1.pdf]

Table S1. GenBank accession numbers. List of GenBank accession numbers corresponding to the ORFV sequences obtained in the present study for the three genes analysed among Sardinian viral samples. Lesion severity is expressed in S (severe) and M (mild).

Sampling code	B2L	VIR	O45	Lesion severity	Host
ORF3_SS_2017	MW043017	MW043053	MW043035	S	Goat
ORF4_SS_2017	MW043018	MW043054	MW043036	S	Goat
ORF5_SS_2017	MW043019	MW043055	MW043037	S	Goat
ORF10_SS_2019	MW043021	MW043057	MW043039	M	Goat
ORF11_SS_2019	MW043020	MW043056	MW043038	M	Goat
ORF12_SS_2019	MW043022	MW043058	MW043040	M	Goat
ORF13_SS_2019	MW043023	MW043059	MW043041	M	Goat
ORF14_SS_2019	MW043024	MW043060	MW043042	M	Goat
ORF6_SS_2017	MW043025	MW043061	MW043043	M	Sheep
ORF7_SS_2017	MW043026	MW043062	MW043044	M	Sheep
ORF1_SS_2017	MW043027	MW043063	MW043045	S	Sheep
ORF2_SS_2017	MW043028	MW043064	MW043046	S	Sheep
ORF16_SS_2019	MW043029	MW043065	MW043047	M	Sheep
ORF8_CA_2018	MW043030	MW043066	MW043048	S	Sheep
ORF9_CA_2018	MW043033	MW043069	MW043051	S	Sheep
ORF17_SS_2019	MW043032	MW043068	MW043050	M	Sheep
ORF15_SS_2019	MW043034	MW043070	MW043052	M	Sheep
ORF18_SS_2019	MW043031	MW043067	MW043049	M	Man
